# Effects of Mulberry Leaf and Corn Silk Extracts Against α-Amylase and α-Glucosidase In Vitro and on Postprandial Glucose in Prediabetic Individuals: A Randomized Crossover Trial

**DOI:** 10.3390/nu17213438

**Published:** 2025-10-31

**Authors:** You Sun, Xiaokang Niu, Yifan Wang, Qi Zhang, Yan Liu, Jingjing He, Lingling Xu, Ran Wang, Jie Guo

**Affiliations:** 1 College of Food Science and Engineering, Tianjin University of Science and Technology, Tianjin 300457, China; 2 Key Laboratory of Precision Nutrition and Food Quality, Department of Nutrition and Health, China Agricultural University, Beijing 100080, China; 3 National Center of Technology Innovation for Dairy, Hohhot 010110, China; 4 Key Laboratory of Endocrinology of National Health Commission, Department of Endocrinology, Peking Union Medical College Hospital, Chinese Academy of Medical Science, Beijing 100730, China

**Keywords:** enzymes, prediabetes, postprandial glucose, herbal extract, milk

## Abstract

Objective: Postprandial hyperglycemia is a major risk factor for type 2 diabetes and cardiovascular disease. Inhibition of α-amylase and α-glucosidase can attenuate postprandial glycemic response (PPGR). This study aimed to investigate the inhibitory effects of mulberry leaf and corn silk on these enzymes in vitro and their impact on postprandial glucose (PG) levels in prediabetic individuals using milk-based matrices. Research Design and Methods: In vitro, enzyme inhibition was assessed using the DNS method (α-amylase) and pNPG method (α-glucosidase). A randomized crossover trial was conducted in 11 prediabetic individuals with four interventions: pure milk; lactose-hydrolyzed milk; lactose-hydrolyzed milk with mulberry leaf, corn silk, and resistant dextrin; and GOS milk with mulberry leaf and corn silk. PPGR was assessed by area under the glucose curve, 1 and 2 h PG, maximum PG, and 2 h glucose excursion. Paired Wilcoxon signed-rank tests were used for comparisons. Results: Mulberry leaf and corn silk extracts inhibited both enzymes dose-dependently, with synergistic effects. No significant differences in PPGR indices were observed across interventions in the overall prediabetic individuals. However, in the overweight subgroup, the combination of GOS milk supplemented with mulberry leaf and corn silk significantly reduced 1 h PG (median difference [P25, P75]: −0.84 mmol/L [−1.05, −0.49]), maximum PG (−0.54 mmol/L [−0.75, −0.25]), and glucose excursion (−0.62 mmol/L [−0.75, −0.24]) compared to pure milk. Conclusions: Mulberry leaf and corn silk extracts inhibit α-amylase and α-glucosidase in vitro and may attenuate postprandial glucose excursions in overweight prediabetic individuals when delivered in a GOS milk matrix.

## 1. Introduction

Postprandial hyperglycemic response is not only a hallmark of prediabetes and type 2 diabetes but also constitutes an independent risk factor distinct from fasting blood glucose (FBG) and HbA1c. It demonstrates significant associations with type 2 diabetes progression, incident cardiovascular disease (CVD), and elevated all-cause mortality risk [1]. The escalating burden of prediabetes, a precursor to diabetes, underscores the urgency of addressing postprandial glucose management. According to the International Diabetes Federation 2021 report, the prevalence of impaired glucose tolerance and impaired fasting glucose was 9.1% and 5.8%, respectively. It is expected that these numbers will increase to 10% and 6.5% by 2045, respectively [2]. Around 38.1% of the population in China is in the prediabetes stage, and these people have a significantly higher risk of developing diabetes and its complications [3]. This high prevalence not only poses a significant threat to public health but also forecasts a substantial future economic burden on the healthcare system. A pivotal strategy for controlling postprandial hyperglycemia involves inhibiting the activity of enzymes (α-amylase and α-glucosidase) or modulating glucose transport [4,5]. Conventional pharmaceutical interventions for postprandial hyperglycemia are frequently associated with gastrointestinal adverse effects, including flatulence, diarrhea, and abdominal pain [6]. In contrast, medicinal food homologous substances—by leveraging their natural bioactive constituents (e.g., alkaloids, polysaccharides, polyphenols)—demonstrate clinically relevant glucose control with superior safety profiles in clinical investigations. Mulberry leaf, the dried foliage of Moraceae plants and a canonical “medicinal and edible” herb in China, may regulate postprandial glucose through two mechanisms: (1) direct inhibition of α-amylase and α-glucosidase activity via flavonoids that competitively block substrate binding and catalytic processes through hydrogen bonding and hydrophobic interactions at the enzymes’ active sites, thereby retarding carbohydrate hydrolysis; and (2) downregulation of mRNA expression for intestinal glucose transporters sodium-glucose cotransporter 1 (SGLT1) and glucose transporter 2 (GLUT2), consequently inhibiting enterocytic glucose translocation into systemic circulation [4,7,8,9,10]. Corn silk, comprising the dried stigmas and styles of the pistillate flowers from *Zea mays* L. (Poaceae family), is recognized as a medicinal food homologous substance [11]. In vitro studies have confirmed its inhibitory effects on α-amylase and α-glucosidase activity, wherein corn silk flavonoids competitively occupy the enzymes’ active sites through hydrogen bonding and π-π interactions, sterically hindering substrate binding to α-glucosidase and consequently retarding carbohydrate hydrolysis [12,13]. Detailed information on the pharmacological profiles of mulberry leaf and corn silk extracts—including their key phytochemical components, molecular structures, molecular formulas, and documented metabolic health benefits—is summarized in Supplementary Table S1.

Compared to individual bioactive compounds, combinatorial formulations leverage synergistic effects to enhance functional potency, reduce dosage requirements, and mitigate toxicity risks. Building upon the complementary advantages of mulberry leaf and corn silk extracts, this study aimed to investigate their in vitro synergistic inhibition of α-amylase and α-glucosidase. Milk, as a pivotal nutritional vehicle in human dietary systems, offers high biocompatibility and frequent consumption patterns, making it an ideal delivery matrix for homologous phytochemicals in medicinal foods [14]. Nevertheless, China’s current dairy intake (42.6 kg/capita/year) falls significantly below recommended levels (112.8 kg/year), attaining only 37.8% of dietary guidelines [15]. This gap may correlate with the high prevalence of lactase deficiency [16]. Affected individuals typically experience abdominal bloating, diarrhea, and other gastrointestinal distress symptoms following conventional milk consumption, directly suppressing dairy intake. To address this, the lactose-free dairy market continues expanding [17], with two technologically distinct products being particularly relevant: (1) Lactose-hydrolyzed milk functional dairy product wherein β-galactosidase pre-hydrolyzes lactose into glucose and galactose [18]; and (2) GOS-enriched milk, produced via β-galactosidase-mediated transgalactosylation that catalyzes intermolecular galactosyl transfer, generating milk intrinsically fortified with prebiotic galacto-oligosaccharides (GOS) [19]. Employing a randomized crossover trial design in a prediabetic cohort, we systematically compare the postprandial glucose impact of four milk matrices: (i) lactose-hydrolyzed milk; (ii) pure milk; (iii) lactose-hydrolyzed milk supplemented with mulberry leaf and corn silk extracts and resistant dextrin; and (iv) GOS-enriched milk containing mulberry leaf and corn silk extracts, thereby providing evidence-based dietary strategies for prediabetes management.

## 2. Materials and Methods

### 2.1. In Vitro Experiments

#### 2.1.1. Chemicals and Reagents

Porcine pancreatic α-amylase, soluble starch, sodium phosphate buffer, p-nitrophenyl-α-D-glucopyranoside (pNPG), α-glucosidase, and anhydrous sodium carbonate were purchased from Yuanye Bio-Technology Co., Ltd., Shanghai, China. DNS reagent was obtained from Solarbio Science & Technology Co., Ltd., Beijing, China. Acarbose tablets were purchased from a pharmacy. Mulberry leaf and corn silk extracts were provided by Guangdong Qingyunshan Pharmaceutical Co., Ltd., Shaoguan, China. Resistant dextrin was provided by Baolingbao Biotechnology Co., Ltd., Dezhou, China. Among them, mulberry leaf and corn silk extracts were prepared by water extraction. The quantification results of bioactive compounds (e.g., flavonoid content) in mulberry leaf and corn silk extracts are presented in Supplementary Table S2.

#### 2.1.2. α-Amylase Inhibitory Assay

A slightly modified α-amylase inhibition rate assay was adopted [20,21]. The detailed reagent volumes and procedural steps for the Control group, Blank group, Experimental group, and Experimental blank group in this α-amylase inhibitory activity assay are presented in Supplementary Table S3. Briefly, 50 μL of mulberry leaf or corn silk extracts at different concentrations were added to 150 μL of soluble starch solution. The reaction was initiated by adding 50 μL of α-amylase (10.4 U/mL in 0.1 M sodium phosphate buffer, pH 6.9) to the mixture, followed by incubation at 37 °C. After 30 min, the reaction was terminated by adding 20 μL of 2 M NaOH solution. Subsequently, 20 μL of DNS reagent was added to the reaction mixture, and the mixture was subjected to a boiling water bath for 20 min. The absorbance at a wavelength of 540 nm was measured using a microplate reader. The α-amylase inhibition experiment was performed according to the group design and operational details specified in Supplementary Table S3, and the α-amylase inhibition rate was calculated using the following Equation (1). First, background interference (arising from the intrinsic reaction system and sample color) is subtracted from the Experimental group, leaving absorbance attributable solely to α-amylase–catalyzed starch hydrolysis in the presence of the sample. Likewise, background interference is removed from the Control group, yielding absorbance corresponding to α-amylase–mediated hydrolysis without inhibition. The ratio of these values reflects the residual α-amylase activity in the Experimental group relative to the Control. Subtracting this ratio from 1 and multiplying by 100% gives the percentage inhibition of α-amylase activity by the sample.

(1) $$\mathrm{Inhibition}\;\mathrm{rate}\;(\%)=(1-\frac{{OD}_{A}-{OD}_{a}}{{OD}_{B}-{OD}_{b}})\;\times \;100\%$$

In Equation (1), OD*_A_* is the absorbance value of the Experimental group; OD*_a_* is the absorbance value of the Experimental blank group; OD*_B_* is the absorbance value of the Control group; and OD*_b_* is the absorbance value of the Blank group. We explicitly defined the composition of two blank groups: the Experimental blank group (OD*_a_*) does not contain α-amylase/α-glucosidase but contains plant extracts; the Blank group (OD*_b_*) does not contain plant extracts and α-amylase/α-glucosidase. To ensure volume consistency in each reaction system, the volume of the missing plant extracts or enzymes was replaced with an equal volume of buffer. The intrinsic color of plant extracts (e.g., flavonoids, polyphenols) may cause non-specific absorption at 540/405 nm. Subtracting the blank absorbance ensures that the measured values exclusively reflect the hydrolysis of starch by α-amylase/α-glucosidase, eliminates background interference, and thereby ensures the accuracy of inhibition rate calculations.

#### 2.1.3. α-Glucosidase Inhibitory Assay

A slightly modified α-glucosidase inhibition rate assay was used [22,23]. α-glucosidase (2 U/mL), the substrate p-nitrophenyl-α-D-glucopyranoside (pNPG, 8 mM), and mulberry leaf or corn silk extract solutions were all prepared using PBS (pH 6.8, 0.1 mol/L). 80 μL of sample solutions at different concentrations and 20 μL of α-glucosidase solution were mixed and incubated at 37 °C for 10 min, after which 40 μL of pNPG was added to initiate the reaction. Following a 30 min reaction at 37 °C, 60 μL of 0.1 M Na_2_CO_3_ solution was immediately added to terminate the reaction, and the absorbance at a wavelength of 405 nm was measured using a microplate reader. The α-glucosidase inhibition experiment was conducted with the experimental design the same as that of the α-amylase inhibition experiment and according to Supplementary Table S4, and the α-glucosidase inhibition rate was calculated using Equation (1).

#### 2.1.4. Inhibitory Effect of Mulberry Leaf and Corn Silk Combinations on the Two Enzymes

Using the methods described in detail in the previous two sections, the synergistic effects of the combined extract of mulberry leaf and corn silk on α-amylase and α-glucosidase were studied. Briefly, mulberry leaf and corn silk extracts were combined at varying mass ratios, enzymatic reactions were initiated with substrate addition, inhibitory activities were quantified via the DNS method for α-amylase and the pNPG assay for α-glucosidase, and combination indices (CI) were subsequently calculated according to Chou’s method [24], and the formula for calculating CI values is as follows (Equation (2)).

(2) $$\mathrm{Combination}\;\mathrm{Indices}\;(\mathrm{CI})=(\frac{{\left(D\right)}_{1}}{{\left(Dx\right)}_{1}}\;+\;\frac{{\left(D\right)}_{2}}{{\left(Dx\right)}_{2}})$$

In Equation (2), (D)_1_ is the actual dose of mulberry leaf in combination; (D)_2_ is the actual dose of corn silk in combination; (Dx)_1_ is the concentration of mulberry leaf alone for target effect (when combined with corn silk); and (Dx)_2_ is the concentration of corn silk alone for target effect (when combined with mulberry leaf). The values of (Dx)_1_ and (Dx)_2_ can be calculated using the concentration–enzyme inhibition rate fitting curve equations in Supplementary Figure S1.

### 2.2. In Vivo Experiments

#### 2.2.1. Research Design

Extracts of mulberry leaf and corn silk, along with resistant dextrin, were added to lactose-hydrolyzed milk and galactooligosaccharide (GOS)-fortified milk. A randomized crossover trial was conducted to compare the effects of four different samples (detailed in Supplementary Table S5, which presents their content and production methods) on postprandial glucose levels in individuals with prediabetes. Each participant attended a total of four food trial sessions, with the order of test samples randomized and a 7-day interval between trials. Each session involved acute intake of one test sample and 2 h postprandial glucose monitoring. This aligns with the randomized crossover trial design for evaluating acute postprandial glycemic responses, matching the study’s goal of assessing immediate glucose effects [25]. The trial was carried out at Peking Union Medical College Hospital in Beijing, China, from 30 July 2024, to 11 January 2025. This research protocol was approved by the Institutional Review Board of the Ethics Committee of China Agricultural University (CAUHR-20231206, registered on 15 December 2023) and the Ethics Committee of Peking Union Medical College Hospital (I-24PJ0448, registered on 29 February 2024), and was registered with the Chinese Clinical Trial Registry (ChiCTR2400083330). All participants signed informed consent forms.

#### 2.2.2. Participant Eligibility Criteria

Adults aged 18 years and above who met the diagnostic criteria for prediabetes and had not received antihyperglycemic medication treatment were included. Participants with food allergies or severe lactose intolerance (defined as persistent gastrointestinal symptoms after consuming less than 240 mL of milk) were excluded [26]. Impaired fasting blood glucose and impaired glucose tolerance are collectively referred to as prediabetes. Specifically, impaired fasting blood glucose is defined as a fasting blood glucose level of 5.6–6.9 mmol/L; impaired glucose tolerance is defined as a 2 h postprandial glucose (2 h PG) level of 7.8–11.0 mmol/L; or prediabetes can also be defined as a glycosylated hemoglobin level of 5.7–6.4% [27,28].

#### 2.2.3. Interventions

After an overnight fast (10–12 h), the subjects participated in the food trial. The four intervention samples were as follows: (1) lactose-hydrolyzed milk group: one pack of whole-wheat bread (50 g of carbohydrates) and 220 mL of lactose-hydrolyzed milk; (2) pure milk group: one pack of whole-wheat bread (50 g of carbohydrates) and 220 mL of pure milk; (3) mulberry leaf + corn silk + resistant dextrin + lactose-hydrolyzed milk group: one pack of whole-wheat bread (50 g of carbohydrates) and 220 mL of lactose-hydrolyzed milk supplemented with mulberry leaf, corn silk extracts, and resistant dextrin; (4) mulberry leaf + corn silk + GOS milk group: one pack of whole-wheat bread (50 g of carbohydrates) and 220 mL of GOS milk supplemented with mulberry leaf and corn silk extracts. All four samples were sterilized by Ultra-High-Temperature Instantaneous Sterilization (UHT). The order of the four intervention samples for each subject, along with the allocation of their serial numbers, was determined by the study designer using random numbers generated in Excel. All intervention samples were uniformly packaged, with serial numbers printed on the outer packaging; the serial numbers (corresponding to each assigned sample) were enclosed in separate sealed opaque envelopes. On the intervention day, participants received the envelopes, and throughout the intervention, both participants and implementers were unaware of the specific sample identity, ensuring a double-blind design. Statistical analysis was performed by researchers who remained blinded. During the trial (2 h), subjects could only consume the provided intervention samples and were not allowed to eat any other food.

#### 2.2.4. Measurement

Data on sociodemographic factors (age, gender, and education level), lifestyle factors (body mass index [BMI], smoking status, and drinking status), and family history of diabetes were collected via questionnaires. BMI was further categorized into four groups: underweight (<18.5 kg/m^2^), normal weight (18.5–24.0 kg/m^2^), overweight (24.0–28.0 kg/m^2^), and obese (≥28.0 kg/m^2^) [29]. During the follow-up period, venous blood samples were collected from the subjects by medical staff at fasting (t = 0 min) and 30, 60, 90, and 120 min after meal intake, and glucose levels at different time points were measured by a testing company. Consequently, metrics related to postprandial glycemic response (PPGR) were obtained. The primary outcome of the study was the area under the glucose curve (AUC) within 2 h after intervention. Secondary outcomes included 1 h postprandial glucose (1 h PG), 2 h PG, maximum glucose, and maximum glucose excursion from baseline.

#### 2.2.5. Sample Size Calculation

The sample size calculation was based on a randomized crossover trial, with the area under the glucose curve as the primary outcome measure [30]. Using the nonparametric Wilcoxon signed-rank test to analyze the intra-group differences in the primary outcome variable, it was determined that a 20% difference in the mean postprandial glucose, measured by AUC, with α = 0.05 and β = 0.2, and accounting for a 10% dropout rate, would require a total of 13 participants.

### 2.3. Statistical Analysis

All in vitro experiments were performed in three independent replicates, and the results are expressed as the mean ± standard deviation (SD). For the human trial part, the paired Wilcoxon signed-rank test was used to compare differences in AUC, 1 h PG, 2 h PG, maximum glucose, and maximum glucose excursion from baseline among different intervention samples. Analyses were conducted using SPSS (version 27.0). GraphPad Prism 9 was used to plot. *p*-values < 0.05 were considered statistically significant.

## 3. Results

### 3.1. Inhibitory Activity of Mulberry Leaf Extracts on α-Amylase and α-Glucosidase

**Figure 1:**
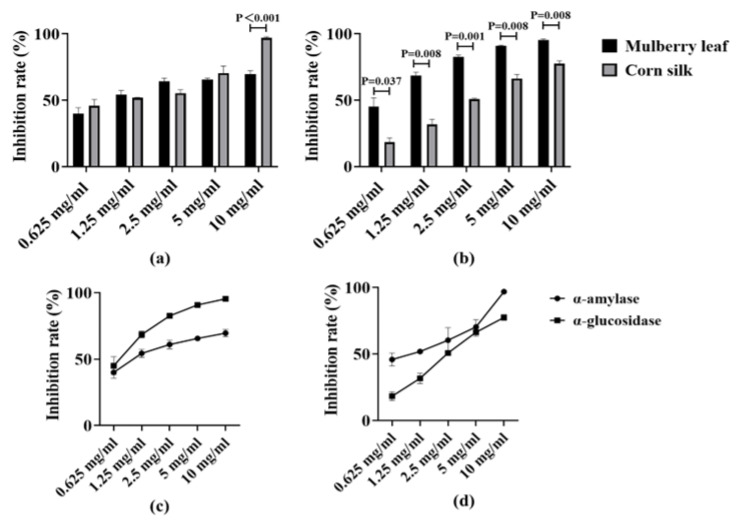
Inhibition rates of extracts at different concentrations on α-amylase and α-glucosidase. (**a**) Comparison of the inhibition rates of mulberry leaf and corn silk on α-amylase at the same concentration; (**b**) Comparison of the inhibition rates of mulberry leaf and corn silk on α-glucosidase at the same concentration; (**c**) Line chart of inhibition rates of mulberry leaf extracts at different concentrations on α-amylase and α-glucosidase; (**d**) Line chart of inhibition rates of corn silk extracts at different concentrations on α-amylase and α-glucosidase.

Figure 1a,b respectively compare the inhibition rates of mulberry leaf and corn silk at different concentrations on α-amylase and α-glucosidase, and there are significant differences in inhibition rates between them at most concentrations (*p* < 0.05). The experimental results are shown in Figure 1c. The acarbose group at a concentration of 0.625 mg/mL was used as the positive control, which exhibited high inhibitory rates against both α-amylase and α-glucosidase (63% and 99.1% respectively). Different concentrations of mulberry leaf (0.625–10 mg/mL) exerted distinct inhibitory effects on the two enzymes. For α-amylase, the inhibition rates of mulberry leaf across various concentrations were relatively stable, mostly around 60%. For α-glucosidase, the inhibition rate varied with changes in the concentration of mulberry leaf. Overall, at most concentrations, its inhibition rate was higher than that against α-amylase, and could reach 70–90% at certain concentrations (e.g., 5 mg/mL, 10 mg/mL, etc.). At high concentrations (5–10 mg/mL), its inhibitory rate on the enzyme is comparable to that of acarbose.

### 3.2. Inhibitory Activity of Corn Silk Extracts on α-Amylase and α-Glucosidase

The experimental results are shown in Figure 1d. For α-amylase, the inhibitory rate of corn silk exhibits an upward trend with increasing concentration, reaching approximately 50% at low concentrations (0.625–1.25 mg/mL), rising to close to 60% at medium concentrations, and significantly increasing at high concentrations. For α-glucosidase, its inhibitory rate also increases with the rise in concentration, being relatively low at low concentrations with a gentle increasing trend, and exceeding 60% and 80% respectively at high concentrations (5–10 mg/mL). Among them, the inhibitory rate of corn silk on α-amylase at high concentrations exceeds that of the positive control acarbose, and the inhibitory rate on α-glucosidase is also close to the level of acarbose.

### 3.3. Inhibitory Effects of Mulberry Leaf and Corn Silk Extracts Combination on α-Amylase and α-Glucosidase

The inhibitory effects of the mixture on α-amylase and α-glucosidase were evaluated by mixing mulberry leaf and corn silk extracts at different concentrations. Nine combinations were obtained by mixing the approximate values of IC_25_ (0.38 mg/mL), IC_50_ (1.13 mg/mL), and IC_75_ (14.06 mg/mL) of mulberry leaf extracts for α-amylase inhibition with the approximate values of IC_25_ (0.27 mg/mL), IC_50_ (1.06 mg/mL), and IC_75_ (5.88 mg/mL) of corn silk extracts for α-amylase inhibition, respectively. Another nine combinations were obtained by mixing the approximate values of IC_25_ (0.29 mg/mL), IC_50_ (0.71 mg/mL), and IC_75_ (1.72 mg/mL) of mulberry leaf extracts for α-glucosidase inhibition with the approximate values of IC_25_ (0.87 mg/mL), IC_50_ (2.61 mg/mL), and IC_75_ (7.83 mg/mL) of corn silk extracts for α-glucosidase inhibition, respectively. The above values can be calculated using the concentration–enzyme inhibition rate fitting curve equations in Supplementary Figure S1. Then, the combination index (CI) was generated based on the in vitro experimental results (Table 1). According to the research on drug synergism, CI < 1, CI = 1, and CI > 1 indicate synergism, additive effect, and antagonism, respectively [24].

When mulberry leaf and corn silk extracts are used in combination, they exhibit a synergistic inhibitory effect on α-amylase, with a CI value < 1. Meanwhile, their combined use also shows a synergistic effect on the inhibition of α-glucosidase, with a CI value < 1. This synergistic effect can reduce the concentration of the extracts used while maintaining or even increasing the enzyme inhibition rate, thereby reducing the release of glucose into the bloodstream and alleviating postprandial glucose.

### 3.4. Impact of Milk Supplemented with Mulberry Leaf, Corn Silk Extracts, and Resistant Dextrin on Postprandial Glucose

A total of 13 prediabetic individuals were enrolled in the study, among whom 11 (84.6%) completed the randomized crossover trial with four samples (Supplementary Figure S2). The mean age of participants was 54 ± 9 years, with ten females, ten having senior high school or higher education, and eight having overweight or obesity (Table 2).

**Figure 2:**
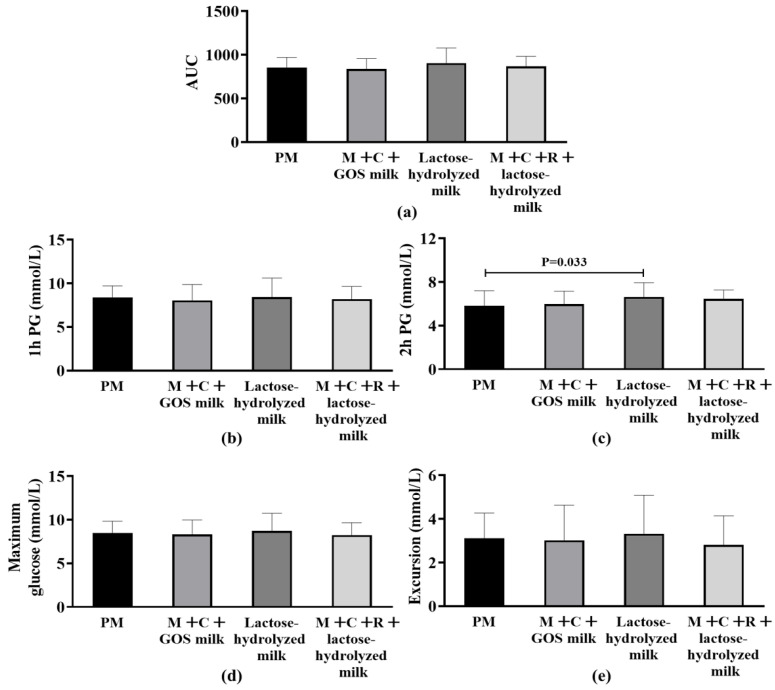
Differences in postprandial glycemic response (PPGR) among four interventions. (**a**) Area Under the Curve (AUC) of postprandial glucose. (**b**) 1 h postprandial glucose (1 h PG). (**c**) 2 h postprandial glucose (2 h PG); statistical significance (*p* = 0.033) is indicated between groups. (**d**) Maximum postprandial glucose concentration. (**e**) Glucose excursion (range of postprandial glucose change). Abbreviation: AUC, Area Under the Curve; PM, pure milk. M + C + GOS milk, mulberry leaf + corn silk + GOS milk; M + C + R + lactose-hydrolyzed milk, mulberry leaf + corn silk + resistant dextrin + lactose-hydrolyzed milk; PG, postprandial glucose.

Figure 2a shows the effects of different interventions on AUC after the subjects consumed 50 g of carbohydrates. The results showed no statistically significant difference in the AUC index among the four groups (*p* > 0.05). Figure 2b–e presents and compares the differences in secondary outcomes among different intervention groups. No statistically significant differences were found among the groups in terms of postprandial maximum glucose, maximum glucose excursion from baseline, and 1 h PG (*p* > 0.05). Notably, the results of 2 h PG showed that the pure milk group was significantly lower than the lactose-hydrolyzed milk group (Median of difference [P25, P75]: −0.86 mmol/L [−1.39, −0.42], *p* = 0.033).

**Figure 3:**
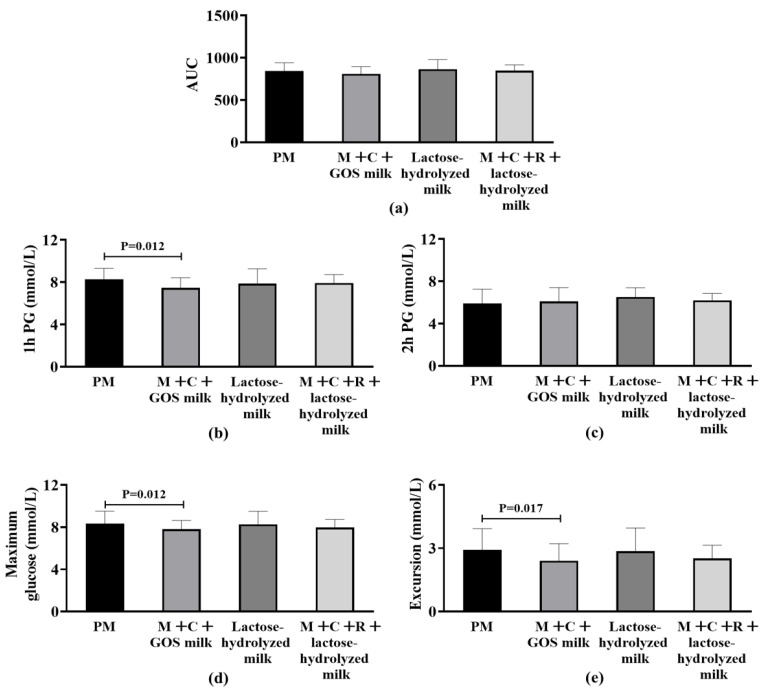
Differences in postprandial glycemic response (PPGR) among overweight subjects (BMI ≥ 24 kg/m^2^) after four intervention. (**a**) Area Under the Curve (AUC) of postprandial glucose. (**b**) 1 h postprandial glucose (1 h PG); statistical significance (*p* = 0.012) is indicated between groups. (**c**) 2 h postprandial glucose (2 h PG). (**d**) Maximum postprandial glucose concentration; statistical significance (*p* = 0.012) is indicated between groups. (**e**) Glucose excursion (range of postprandial glucose change); statistical significance (*p* = 0.017) is indicated between groups. Abbreviation: AUC, Area Under the Curve; PM, pure milk; M + C + GOS milk, mulberry leaf + corn silk + GOS milk; M + C + R + lactose-hydrolyzed milk, mulberry leaf + corn silk + resistant dextrin + lactose-hydrolyzed milk; PG, postprandial glucose.

Among participants being overweight (BMI ≥ 24 kg/m^2^), no statistically significant differences were observed in AUC among the four groups (*p* > 0.05) (Figure 3a). Figure 3b–e shows the differences in secondary outcome indicators among the four intervention groups. The 1 h PG in the mulberry leaf + corn silk + GOS milk group was significantly lower than that in the pure milk group (−0.84 mmol/L [−1.05, −0.49], *p* = 0.012), representing a 4.2% reduction compared to pure milk alone. For the indicator of 2 h PG, no significant difference was found among the groups after statistical testing. Regarding the postprandial maximum glucose level, the mulberry leaf + corn silk + GOS milk group (BMI ≥ 24 kg/m^2^) demonstrated significantly lower values than the pure milk group (BMI ≥ 24 kg/m^2^) (−0.54 mmol/L [−0.75, −0.25], *p* = 0.012), corresponding to a 1.8% reduction compared to pure milk alone. No significant statistical difference was observed between other groups. For maximum glucose excursion from baseline, the mulberry leaf + corn silk + GOS milk group demonstrated significantly lower values than the pure milk group (BMI ≥ 24 kg/m^2^) (−0.62 mmol/L [−0.75, −0.24], *p* = 0.017), representing a 2.9% reduction compared to pure milk alone, and no statistically significant difference was found in this indicator among other sample groups.

## 4. Discussion

In vitro experiments, both mulberry leaf and corn silk extracts have dose-dependent associations with inhibitory rates against α-amylase and α-glucosidase, with a synergistic inhibitory effect. No significant differences in PPGR indices were observed across interventions in the overall prediabetic individuals, except that the 2 h PG in the pure milk group was significantly lower than that in the lactose-hydrolyzed milk group. Among the overweight population, the mulberry leaf + corn silk + GOS milk group, but not other groups, had lower levels of 1 h PG, maximum glucose, and maximum glucose excursion compared to those of pure milk. This implies that mulberry leaf + corn silk + GOS milk may have certain advantages in regulating these blood glucose-related indicators in the overweight population.

As the concentration of mulberry leaf and corn silk extracts increased, their inhibitory rates against the two enzymes gradually approached and even reached the inhibitory level of acarbose at 0.625 mg/mL to a certain extent. These unmodified plant extracts exhibit substantial enzyme inhibitory activity approaching that of conventional drugs, offering valuable insights due to their natural origin and safety profile. Our results were similar to previous studies [31,32]. Han and Gong et al. demonstrated in in vitro enzyme inhibition assays that both mulberry leaf and corn silk extracts exhibited dose-dependent increases in inhibitory rates against α-amylase and α-glucosidase, with efficacy approaching that of the positive control acarbose. Both mulberry leaf and corn silk can bind to the active sites of α-amylase/α-glucosidase via their flavonoids, exerting competitive inhibition on carbohydrate hydrolysis. Mulberry leaf have a stronger inhibitory effect on α-glucosidase, while corn silk is more potent in inhibiting α-amylase [10,12,13]. It is reasonable that their combination may produce a synergistic inhibitory effect through target complementarity. In this study, we did find that the mulberry leaf and corn silk have a synergistic inhibitory effect on the activities of α-amylase and α-glucosidase.

Although there was no significant difference in the PPGR index between different intervention measures in the overall prediabetic individuals, among those with overweight, the combination of GOS milk supplemented with mulberry leaf and corn silk significantly reduced 1 h PG, maximum PG, and 2 h maximum glucose excursion compared with pure milk. This may be explained by insulin sensitivity. Individuals with overweight (BMI ≥ 24 kg/m^2^) are often accompanied by decreased insulin sensitivity, an increased risk of insulin resistance, and weakened ability to regulate postprandial glucose on their own (such as delayed insulin secretion and reduced glucose uptake by peripheral tissues) [33]. In contrast, individuals with normal weight (BMI < 24 kg/m^2^) have better insulin sensitivity. After postprandial glucose rises, it can be rapidly regulated by insulin, leaving limited additional “hypoglycemic space” for intervention measures. Therefore, the difference between the other group and the pure milk group is difficult to detect among those of normal weight. Meanwhile, compared with people of normal weight, overweight individuals have reduced gut microbiota diversity and an increased Firmicutes/Bacteroidetes ratio. As a prebiotic, GOS can promote the growth of beneficial bacteria (such as Bifidobacteria and Lactobacilli) and be rapidly fermented by Bifidobacteria in the gut to produce short-chain fatty acids (SCFAs) like butyrate. SCFAs stimulate the secretion of Glucagon-like Peptide-1 (GLP-1), thereby indirectly improving insulin sensitivity [34,35].

In lactose-hydrolyzed milk, resistant dextrin was also added. However, different from the mechanism of action of mulberry leaf and corn silk, it does not directly affect enzyme activity. Instead, relying on its special molecular structure and physical properties, it increases the viscosity of chyme to form a physical barrier, thereby hindering contact between enzymes and carbohydrates and reducing postprandial glucose levels [36]. Therefore, in the in vitro enzyme inhibition assay, we did not investigate its effect.

The strengths of this study include investigating the effect of mulberry leaf and corn silk on postprandial glucose from the perspective of an in vitro enzyme inhibition experiment and an in vivo human clinical trial. However, this study also has some limitations. First, the relatively small sample size may compromise the statistical power of results and their generalizability to the broader population; notably, the subgroup analysis for overweight participants was not pre-specified, with an even smaller subgroup sample size (reducing conclusion stability) and no significant differences in the primary outcome (AUC) across groups. Second, only postprandial glucose was measured, lacking data on mechanistic biomarkers such as insulin and GLP-1, which hinders in-depth elaboration of specific glucose-regulating mechanisms. Third, the short intervention duration prevents assessing sustained effects of long-term consumption of such milk on blood glucose and metabolic indicators in prediabetic individuals. Lastly, the study only recruited prediabetic participants, so results should be cautiously generalized to other populations. In the future, it is necessary to verify our findings among studies with larger samples and subgroup designs.

## 5. Conclusions

This study systematically evaluated the effects of mulberry leaf and corn silk extracts, as well as their combinations, on α-amylase and α-glucosidase activities, and postprandial glucose regulation, using both in vitro enzyme inhibition assays and an in vivo human intervention trial. In vitro, mulberry leaf and corn silk extracts demonstrated dose-dependent inhibition of α-amylase and α-glucosidase, supporting their potential as natural enzyme inhibitors with favorable safety profiles. Notably, specific combinations of the two extracts exhibited synergistic effects, enhancing inhibitory activity and suggesting optimized ratios for functional applications. In the human trial, no significant differences in PPGR were observed across all interventions in the overall prediabetic participants. However, in the overweight subgroup (BMI ≥ 24 kg/m^2^), the combination of mulberry leaf, corn silk, and GOS milk significantly improved PPGR compared to pure milk. Notably, the administered doses of mulberry leaf extract and corn silk extract in this short-term intervention were below their respective maximum recommended daily intake levels; as these substances are classified as both food and medicine, they are generally regarded as safe, and no adverse events were reported by participants following sample consumption. These findings suggest that this functional milk formulation may improve postprandial glucose control in overweight individuals with prediabetes, providing a basis for incorporating natural extracts into personalized nutritional strategies for glycemic management.

## Figures and Tables

**Table 1:** Combination study of mulberry leaf and corn silk on the inhibition of the two enzymes.

Experimental Grouping	Mulberry Leaf (mg/mL):Corn Silk (mg/mL)	α-Amylase		Mulberry Leaf (mg/mL):Corn Silk (mg/mL)	α-Glucosidase	
		Inhibition Rate (%)	CI		Inhibition Rate (%)	CI
1	0.38:0.27	49.7	0.601	0.29:0.87	54.1	0.641
2	0.38:1.06	57.6	0.848	0.29:2.61	65.4	0.778
3	0.38:5.88	83.1	0.850	0.29:7.83	85.2	0.623
4	1.13:0.27	60.1	0.539	0.71:0.87	66.1	0.757
5	1.13:1.06	76.0	0.254	0.71:2.61	72.3	0.844
6	1.13:5.88	86.0	0.795	0.71:7.83	87.8	0.623
7	14.06:0.27	84.8	0.277	1.72:0.87	83.1	0.740
8	14.06:1.06	89.4	0.222	1.72:2.61	85	0.773
9	14.06:5.88	90.8	0.753	1.72:7.83	91.6	0.631

Abbreviation: CI, combination index. Both mulberry leaf extract and corn silk extract were used at 0.5 mL per group. Since mass = concentration × volume, the concentration ratio (mulberry leaf: corn silk) in the table equals their mass ratio.

**Table 2:** Characteristics of subjects at baseline.

Characteristics	All (n = 11)
Age (years, mean ± SD)	54 ± 9
Sex (%)	
Male	1 (9.1)
Female	10 (90.9)
Education level, N (%)	
Junior high school and below	1 (9.1)
Senior high school and above	10 (90.9)
BMI (kg/m^2^, mean ± SD)	26.26 ± 4.61
BMI group, N (%)	
Normal weight	3 (27.3)
Overweight	5 (45.4)
Obese	3 (27.3)
Smoking status, N (%)	
Non-current smoker	9 (81.8)
Current smoker	1 (9.1)
Former smoker	1 (9.1)
Drinking status (%)	
Current drinker	1 (9.1)
Non-drinker	10 (90.9)
FBG (mmol/L)	5.47 ± 0.5
Family history of diabetes (%)	
Yes	6 (54.5)
No	5 (45.5)

Abbreviation: SD, standard deviation; BMI, body mass index; FBG, fasting blood glucose. Data were presented as mean ± standard deviation or n (%).

## Data Availability

Data are contained within the article. Further inquiries can be directed to the corresponding author.

## Supplementary Materials

The following supporting information can be downloaded at: https://www.mdpi.com/article/10.3390/nu17213438/s1; Supplementary Table S1. Pharmacological properties table of mulberry leaf and corn silk extracts; Supplementary Table S2. The content of active ingredients in substances of homology of food and medicine; Supplementary Table S3. In vitro α-amylase inhibitory activity assay; Supplementary Table S4. In vitro α-glucosidase inhibitory activity assay; Supplementary Figure S1. The concentration-enzyme inhibition fitting curve of mulberry leaf and corn silk; Supplementary Table S5. Sample information; Supplementary Figure S2. Flow chart.
