# Supplementary material for: Effects of Mulberry Leaf and Corn Silk Extracts Against α-Amylase and α-Glucosidase In Vitro and on Postprandial Glucose in Prediabetic Individuals: A Randomized Crossover Trial
Source: Nutrients. 2025 Oct 31;17(21):3438. doi: 10.3390/nu17213438 (PMC12610157; doi:10.3390/nu17213438)
Supplement: Supplementary file 1 [file nutrients-17-03438-s001.zip › nutrients-3900296-supplementary.pdf]

CONSORT 2010 checklist of information to include when reporting a randomised trial*

Section/Topic	Item No	Checklist item	Reported on page No
**Title and abstract**			
	1a	Identification as a randomised trial in the title	1
	1b	Structured summary of trial design, methods, results, and conclusions (for specific guidance see CONSORT for abstracts)	1
**Introduction**			
Background and objectives	2a	Scientific background and explanation of rationale	2
	2b	Specific objectives or hypotheses	2
**Methods**			
Trial design	3a	Description of trial design (such as parallel, factorial) including allocation ratio	4
	3b	Important changes to methods after trial commencement (such as eligibility criteria), with reasons	NA
Participants	4a	Eligibility criteria for participants	5
	4b	Settings and locations where the data were collected	5
Interventions	5	The interventions for each group with sufficient details to allow replication, including how and when they were actually administered	5
Outcomes	6a	Completely defined pre-specified primary and secondary outcome measures, including how and when they were assessed	6
	6b	Any changes to trial outcomes after the trial commenced, with reasons	NA
Sample size	7a	How sample size was determined	6
	7b	When applicable, explanation of any interim analyses and stopping guidelines	NA
Randomisation:			
Sequence generation	8a	Method used to generate the random allocation sequence	5
	8b	Type of randomisation; details of any restriction (such as blocking and block size)	5
Allocation concealment mechanism	9	Mechanism used to implement the random allocation sequence (such as sequentially numbered containers), describing any steps taken to conceal the sequence until interventions were assigned	5
Implementation	10	Who generated the random allocation sequence, who enrolled participants, and who assigned participants to interventions	14
Blinding	11a	If done, who was blinded after assignment to interventions (for example, participants, care providers, those assessing outcomes) and how	5
	11b	If relevant, description of the similarity of interventions	5

Statistical methods	12a	Statistical methods used to compare groups for primary and secondary outcomes	6
	12b	Methods for additional analyses, such as subgroup analyses and adjusted analyses	6
**Results**			
Participant flow (a diagram is strongly recommended)	13a	For each group, the numbers of participants who were randomly assigned, received intended treatment, and were analysed for the primary outcome	8
	13b	For each group, losses and exclusions after randomisation, together with reasons	8
Recruitment	14a	Dates defining the periods of recruitment and follow-up	4
	14b	Why the trial ended or was stopped	4
Baseline data	15	A table showing baseline demographic and clinical characteristics for each group	9
Numbers analysed	16	For each group, number of participants (denominator) included in each analysis and whether the analysis was by original assigned groups	8
Outcomes and estimation	17a	For each primary and secondary outcome, results for each group, and the estimated effect size and its precision (such as 95% confidence interval)	8
	17b	For binary outcomes, presentation of both absolute and relative effect sizes is recommended	NA
Ancillary analyses	18	Results of any other analyses performed, including subgroup analyses and adjusted analyses, distinguishing pre-specified from exploratory	8
Harms	19	All important harms or unintended effects in each group (for specific guidance see CONSORT for harms)	NA
**Discussion**			
Limitations	20	Trial limitations, addressing sources of potential bias, imprecision, and, if relevant, multiplicity of analyses	12
Generalisability	21	Generalisability (external validity, applicability) of the trial findings	11
Interpretation	22	Interpretation consistent with results, balancing benefits and harms, and considering other relevant evidence	11
**Other information**			
Registration	23	Registration number and name of trial registry	4
Protocol	24	Where the full trial protocol can be accessed, if available	4
Funding	25	Sources of funding and other support (such as supply of drugs), role of funders	14

Supplementary Table S1. Pharmacological properties table of mulberry leaf and corn silk extracts

Bioactive Compound	Molecular Structure (Representative)	Molecular Formula	Relevant Health Benefits for Metabolic Health	References
**Mulberry Leaf Extract**				
**1-Deoxynojirimycin (DNJ)**	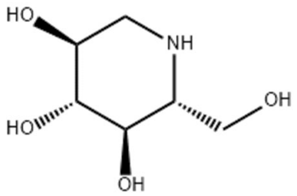	$C_6H_{13}NO_4$	**Potent α-Glucosidase Inhibition:** Delays carbohydrate digestion, reducing postprandial blood glucose levels. **Anti-Diabetic:** Improves glycemic control and insulin sensitivity. **Anti-Obesity:** May reduce body weight gain and lipid accumulation.	Wang H, et al. *Curr Med Chem*. 2021
**Rutin**	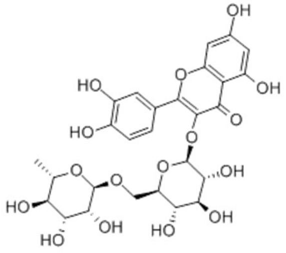	$C_{27}H_{30}O_{16}$	**Antioxidant:** Scavenges free radicals, reducing oxidative stress associated with diabetes and metabolic syndrome. **Anti-inflammatory:** Modulates inflammatory pathways (e.g., NF-κB). **Vascular Protection:** Improves capillary permeability and may lower blood pressure.	Ghorbani A. *Biomed Pharmacother*. 2017
**Isoquercitrin**	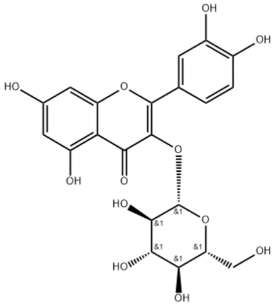	$C_{21}H_{20}O_{12}$	**Enhanced Bioavailability:** More readily absorbed than Quercetin. **Synergistic α-Glucosidase Inhibition:** Works with DNJ to lower blood sugar. **Hepatoprotective & Lipid-Lowering:** Reduces liver fat accumulation and improves lipid profiles.	Valentová K, et al. *Food and Chemical Toxicology*. 2014

**Moracin M**	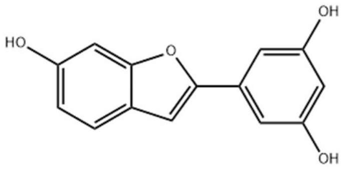	$C_{19}H_{18}O_4$	**PPAR-γ Agonist:** Promotes adipocyte differentiation and improves insulin sensitivity. **Anti-inflammatory:** Inhibits the production of pro-inflammatory cytokines. **Aldose Reductase Inhibition:** May prevent diabetic complications like neuropathy.	Ouassou H, et al. *Med Chem*. 2024
**Corn Silk Extract**				
**Maysin**	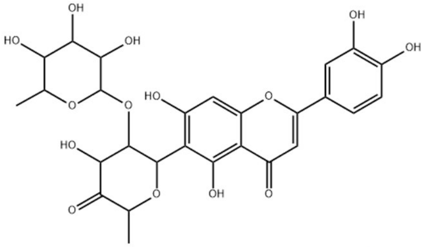	$C_{28}H_{32}O_{16}$	**Potent Antioxidant:** Strong free radical scavenging activity protects pancreatic β-cells and other tissues from oxidative damage. **Anti-hyperglycemic:** Helps lower blood glucose levels through mechanisms potentially involving insulin secretion or sensitivity.	Landeros Martínez LL, et al. *Molecules*. 2023
**Polysaccharides**	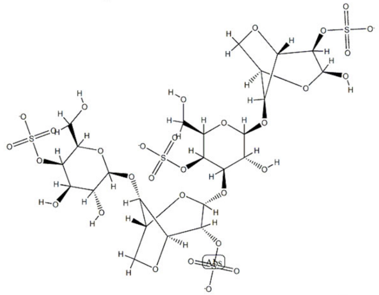	$(C_6H_{10}O_5)_n$	**Diuretic Activity:** Promotes urine flow, aiding in the excretion of excess water, salts, and potentially urea, beneficial for hypertension and edema. **Immunomodulatory:** Enhances immune function. **Prebiotic Effect:** Supports beneficial gut microbiota, which is linked to improved metabolic health.	Zhang Z, et al. *International Journal of Biological Macromolecules*. 2024
**β-Sitosterol**	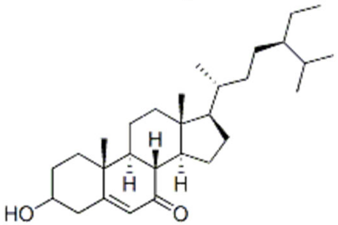	$C_{29}H_{50}O$	**Cholesterol-Lowering:** Competes with dietary cholesterol for absorption, reducing serum LDL-C levels. **Anti-inflammatory:** Modulates immune response to reduce inflammation. **Potential Anti-Diabetic Effect:** May improve insulin	Babu S, et al. *Biomedicine & Pharmacotherapy*. 2020

			signaling.	
**Chlorogenic Acid**	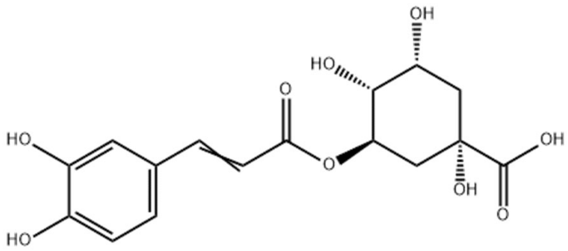	$C_{16}H_{18}O_9$	**Glucose-6-Phosphatase Inhibition:** Reduces hepatic glucose production.<br>**Antioxidant & Anti-inflammatory:** Protects against metabolic oxidative stress.<br>**Improves Endothelial Function:** Beneficial for cardiovascular health in metabolic syndrome.	Tajik N, et al. *Eur J Nutr.* 2017

Supplementary Table S2. The content of active ingredients in substances of homology of food and medicine

**Substances of food and medicine homology**	**Main active ingredient**	**Content**
Mulberry leaf	1-DNJ	1.0%
Corn silk	Crude Polysaccharides	1.0%
	Total Flavonoids	0.1%
Resistant Dextrin	Dietary Fiber	90.0%

Supplementary Table S3. In vitro α-amylase inhibitory activity assay

Reagent	Control group	Blank group	Experimental group	Experimental blank group
10.4 U/ml α-amylase solution	50 μl	0	50 μl	0
Inhibitor	0	0	50 μl	50 μl
Sodium phosphate buffer (pH 6.9, 0.1M)	50 μl	100 μl	0	50 μl
1% Starch solution	150 μl	150 μl	150 μl	150 μl
Incubated in a 37°C water bath for 30 minutes				
20 μl NaOH（2M）solution				
DNS	20 μl	20 μl	20 μl	20 μl
After incubating in a boiling water bath for 20 minutes, it was cooled to room temperature, and the absorbance was measured at 540 nm				

Supplementary Table S4. In vitro α-glucosidase inhibitory activity assay

Reagent	Control group	Blank group	Experimental group	Experimental blank group
Sodium phosphate buffer (pH 6.8, 0.1M)	80 μl	100 μl	0	20 μl
Inhibitor	0	0	80 μl	80 μl
2 U/ml α-glucosidase	20 μl	0	20 μl	0
Incubated at 37°C for 10 minutes				
8 mM pNPG	40 μl	40 μl	40 μl	40 μl
Incubated in a 37°C water bath for 30 minutes, then 60 μl of 0.1 M Na_2CO_3 solution was added to terminate the reaction, and the absorbance was measured at 405 nm				

Supplementary Table S5. Sample information

Sample	Content	Production method
Lactose-hydrolyzed milk	Raw milk, lactase	Under the action of lactase, lactose in milk is broken down into glucose and galactose
Pure milk	Raw milk	
Mulberry leaf + Corn silk + Resistant dextrin + lactose-hydrolyzed milk	Raw milk、lactase、mulberry leaf extracts (110mg/100ml)、corn silk extracts (10mg/100ml)、resistant dextrin (1.5g/100ml)	Under the action of lactase, lactose in milk is broken down into glucose and galactose, with the addition of mulberry leaf, corn silk extracts, and resistant dextrin
Mulberry leaf + Corn silk + GOS milk	Raw milk、lactase、mulberry leaf extracts (110mg/100ml)、corn silk extracts (10mg/100ml)	Under the action of lactase, GOS are generated through lactase transglycosylation, with the addition of mulberry leaf and corn silk extracts

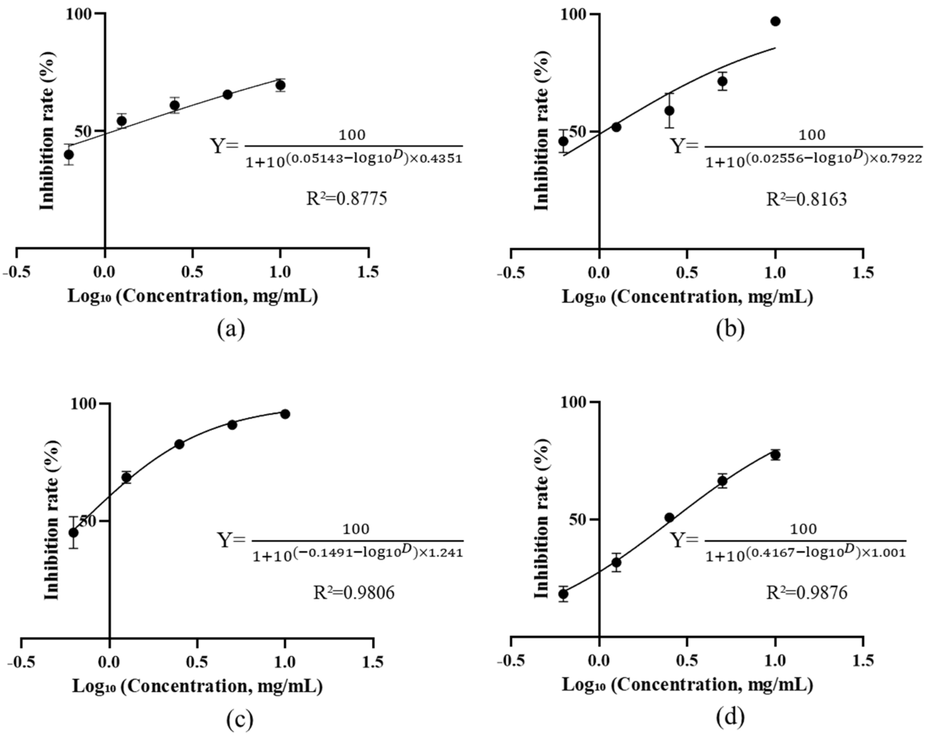

Supplementary Figure S1. The concentration-enzyme inhibition fitting curve of mulberry leaf and corn silk

(a) The inhibition fitting curve of mulberry leaf on α-amylase. **(b)** The inhibition fitting curve of corn silk on α-amylase. **(c)** The inhibition fitting curve of mulberry leaf on α-glucosidase. **(d)** The inhibition fitting curve of corn silk on α-glucosidase.

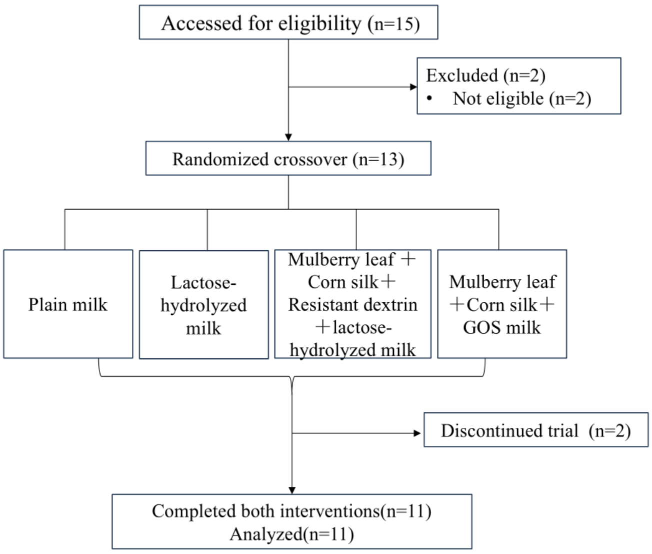

Supplementary Figure S2. Flow chart
